# Copper-64 Dichloride as Theranostic Agent for Glioblastoma Multiforme: A Preclinical Study

**DOI:** 10.1155/2015/129764

**Published:** 2015-11-16

**Authors:** Cristina Ferrari, Artor Niccoli Asabella, Carlo Villano, Beatrice Giacobbi, Daniela Coccetti, Paola Panichelli, Giuseppe Rubini

**Affiliations:** ^1^Nuclear Medicine, University “Aldo Moro”, Piazza Giulio Cesare 11, 70124 Bari, Italy; ^2^Nuclear Medicine, Hospital “Spirito Santo”, Pescara, Italy; ^3^Advanced Center Oncology Macerata (ACOM) S.p.A., Montecosaro, Italy

## Abstract

Glioblastoma multiforme (GBM) is the most common primary malignant brain tumor in adults with a median survival time less than one year. To date, there are only a limited number of effective agents available for GBM therapy and this does not seem to add much survival advantage over the conventional approach based on surgery and radiotherapy. Therefore, the development of novel therapeutic approaches to GBM is essential and those based on radionuclide therapy could be of significant clinical impact. Experimental evidence has clearly demonstrated that cancer cells have a particularly high fractional content of copper inside the nucleus compared to normal cells. This behavior can be conveniently exploited both for diagnosis and for delivering therapeutic payloads (theranostic) of the radionuclide copper-64 into the nucleus of cancerous cells by intravenous administration of its simplest chemical form as dichloride salt [^64^Cu]CuCl_2_. To evaluate the potential theranostic role of [^64^Cu]CuCl_2_ in GBM, the present work reports results from a preclinical study carried out in a xenografted GBM tumor mouse model. Biodistribution data of this new agent were collected using a small-animal PET tomograph. Subsequently, groups of tumor implanted nude mice were treated with [^64^Cu]CuCl_2_ to simulate single- and multiple-dose therapy protocols, and results were analyzed to estimate therapeutic efficacy.

## 1. Introduction

Neoplasms of the central nervous system are one of the leading causes of cancer-related deaths. In particular, glioblastoma multiforme (GBM) is the most common primary malignant brain tumor in adults with median survival time less than one-year and two-year survival rates of 3.3% [1]. To date, only few therapeutic options are available for GBM and their efficacy is limited. Due to poor blood-brain barrier penetration and high-level of GMB chemoresistance, current chemotherapeutic drugs do not add much survival advantage over the classical approach of surgery followed by radiotherapy. Therefore, the investigation of novel therapeutic tools is of crucial importance for the development of more effective GBM treatments.

Based on the evidence that the human copper transporter 1 (CTR1) is overexpressed in a variety of cancers, previous works have consistently demonstrated that copper-64 is an authentic tracer for in vivo characterization of copper metabolism in neoplastic tissues of patients affected by metastatic disease spread out of a variety of tumors, including breast and prostate cancers and malignant cutaneous melanoma, by using noninvasive imaging of positron emission tomography (PET) [2–8].

Literature reported that very sophisticated molecular carriers for ^64^Cu, like monoclonal antibodies, peptides, and nanoparticles, have been developed for combining diagnosis and therapy (theranostic) [9, 10]. However, studies have further demonstrated that these copper complexes have relatively low stability in vivo, rendering the copper prone to reduction and loss from the complex [11].

These results clearly pointed out that chemically much simpler options of ^64^Cu, for example, dichloride salt ([^64^Cu]CuCl_2_), that do not require complicated labeling technologies may exist. It turns out that simple ^64^Cu ions can be employed as theranostic agent by taking advantage of the simultaneous emission of both *β* ^+^ (37.1%) and *β* ^−^ (17.9%) particles (for PET imaging and therapy, resp.) with *T*_1/2_ = 12.7 h. Interestingly, the therapeutic potential of this radionuclide is further enhanced by the decay fraction occurring through electron capture (EC, 43.5%), which stimulates the emission of Auger electrons. Since these particles have relatively low kinetic energies, short-range penetration, but concomitantly high linear energy transfer (LET), they can release high-energy doses (106−109 cGy) within an extremely small volume around the decay site of dimensions of several cubic nanometers. Due to these properties, Auger electrons can manifest radiobiological effects similar to heavier particles [12]. These considerations together with the evidence that cancer cells, unlike normal cells, are enriched in copper content inside the nucleus, lead to predicting that Auger electrons emitted in the very close proximity of the DNA double helix could yield the highest therapeutic effect through the irreversible disruption of the genetic material of cancerous cells [13, 14].

With the purpose of providing preliminary experimental evidence that the above considerations on [^64^Cu]CuCl_2_ can be applied to GBM disease, the present work investigated the biodistribution, tumor uptake, and therapeutic efficacy of different protocols in xenograft U-87MG cell lines implanted in mouse model.

## 2. Materials and Methods

### 2.1. Cell Lines and Growth

U-87MG cell lines were purchased from American Type Culture Collection (ATCC, Manassas, VA). The U-87MG glioblastoma-astrocytoma line was derived from a 44-year-old female. Cell lines were maintained in minimum essential medium Eagle with 2-mM L-glutamine, 0.1-mM nonessential amino acids, 1-mM sodium pyruvate, 100 U/mL penicillin G, and 100 *μ*g/mL streptomycin and supplemented with 10% heat inactivated fetal bovine serum (Sigma, St. Louis, MO). Cells were grown in a humidified atmosphere of 5% CO_2_ in air at 37°C. At the confluence, cells were trypsinized with trypsin-EDTA for use in the following experiments, using the same culture medium. The viability of each group of cells was determined and quantified by Trypan blue exclusion assay.

### 2.2. Subcutaneous Xenograft Mouse Model

Animal studies were approved by local Ethics Committee and conducted according to internationally accepted guidelines. Athymic nu/nu male Balb/c mice were purchased from Charles River Laboratories. They were kept in cages in a separate ventilated/humidified rack at a temperature of 22°C under 12-h day/night cycles and fed ad libitum with standard laboratory food and water. U-87 MG cells were grown routinely as described above. Before injection, cells were trypsinized, collected with media, pelleted by centrifugation at 2000 rpm for 5 min, suspended in phosphate-buffered saline (PBS), and further centrifuged. Cells were finally resuspended in PBS and counted. Using a Hamilton syringe, 50 *μ*L of the cell suspension containing 1 × 10^7^ cells was injected subcutaneously on the back of the neck close to the shoulder in a group of 6-week old ice. Tumors were allowed to growth for 3 weeks before initiating treatment.

### 2.3. Production of Copper-64

Highly enriched ^64^Ni was plated on a gold disk (24 mm in diameter and 2 mm thick) by electrodeposition using a procedure reported previously [14]. Copper-64 was produced by bombardment of the ^64^Ni target in a 18-MeV cyclotron (IBA, Belgium) with a 18-*μ*A proton current at energy of 14.6 MeV. After bombardment, ^64^Cu was separated from the ^64^Ni target and purified from other contaminants by chromatography using an ion-exchange column prepared with a slurry of AGl × 8 anion-exchange resin (Biorad Laboratories, Italy) filled into a glass column 1.0 cm × 30 cm size. Activity was eluted with concentrated HCl (1.0 M) yielding high-specific activity ^64^Cu as anionic copper chloride complexes.

### 2.4. Imaging Studies

Imaging studies of 12 nude mice were performed using a YAPPET micro-PET tomograph (ISE, Pisa, Italy). Mice were anesthetized with a mixture of ketamine (100 mg/kg) and xylazine (7 mg/kg) and placed in supine position under the camera. Biodistribution studies were performed by collecting whole-body scans at 1, 2, 4, and 20 hours after intravenous injection of 12 MBq of [^64^Cu]CuCl_2_ in the tail vein. Acquisition consisted of 128 frames collected over a 360-degree rotation. No attenuation corrections were applied and iterative OSEM 2D algorithm was employed for imaging reconstruction.

To assess tracer distribution, regions of interest (ROIs) were drawn at the tumor, liver, kidneys, and the shoulder opposite to the tumor site using two whole-body frames. Biodistribution of [^64^Cu]CuCl_2_ was assessed by determining tracer uptake semiquantitative parameters measured by maximum standardized uptake value [SUVmax, expressed as ROI uptake (*μ*Ci/mL)/injected dose per unit weight (*μ*Ci/g)]. SUVs determined in tumor, kidneys, and shoulder were normalized to liver uptake and the highest value was displayed.

Statistical analysis of data from semiquantitative PET images was expressed as mean and standard deviation (SD). Multiple paired two-tailed *t*-test was applied to determine significant differences between normalized tracer concentrations observed in tumor, liver, kidneys, and shoulder.

### 2.5. Therapy Studies

Ninety nude mice 9-week old, implanted with U-87 GM cells which allowed to growth tumors to reach at least 1-cm size within 3 weeks, were included in the study. Animals were injected with [^64^Cu]CuCl_2_ by cardiac puncture under anesthesia. They were equally divided into three groups of 30 animals as follows: nontreated (control group, CG), treated with a single administration of 333 MBq of [^64^Cu]CuCl_2_ (single-dose group, SDG), and treated with a multiple-dose regimen of 55.5 MBq × 6 days of [^64^Cu]CuCl_2_, one injection being administered per day for six subsequent days (multiple-dose group, MDG). Serial whole-body micro-PET images were performed at 1 week (PET-1) and 20 weeks after the end of treatment (PET-2) in the treated mice to monitor the tumor volume by a direct calculation of the volume of interest (VOI) estimated semiquantitatively.

The reduction of the tumor volume from PET-1 to PET-2 was expressed as a percentage. Survival rates among the three groups were evaluated by Student's *t*-test for unpaired groups. A *p*-value < 0.005 was considered statistically significant. Statistical evaluation was carried out using SPSS 20.0 for Macintosh.

### 2.6. Inhibition of Neurosphere Formation

U-87MG cell has the tendency to aggregate in serum-containing medium (SCM) to form neurospheres, spherically shaped preorganized structures. Cultured GBM cells were allowed to form neurospheres after 7 days in SCM. Then, aggregated cells were separated by trypsinization and exposed to [^64^Cu]CuCl_2_ for a maximum of 18 h. The radio-treated cells were cultured again at a cell density of 20 cells per well in an ultra-low-attachment 96-well plates (30 wells) containing nonradiation exposed SCM for 14 days.

## 3. Results

### 3.1. Imaging Studies

**Figure 1:**
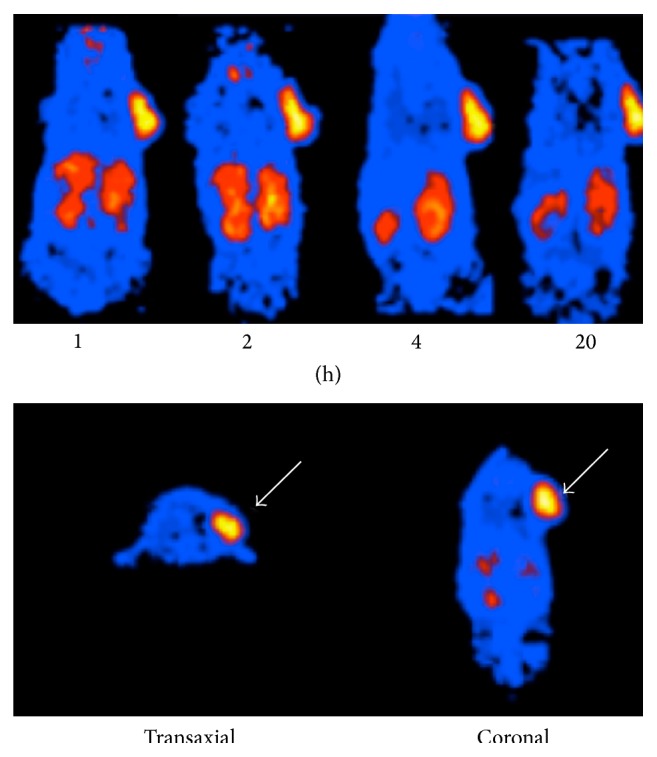
Whole-body mouse biodistribution images collected 1, 2, 4, and 20 hours after intravenous injection of 12 MBq of [^64^Cu]CuCl_2_. Increased tracer uptake is observed in the tumor graft (white arrow).

PET images collected 1, 2, 4, and 20 hours after injection showed a good visualization of tumor grafts in all animals (Figure 1). There was a prominent activity in tumors as compared to the opposite reference shoulder. As expected, whole-body images displayed a high liver uptake 1 hour after injection, followed by intestinal washout 20 hours after injection. No significant uptake was observed in the brain. The mean value of SUVmax (±SD) of GBM grafts was 3.6 ± 0.44 as compared to that in soft tissue (shoulder) that was 1.25 ± 0.14. The mean value of SUVmax in the liver was found to be as high as 20.3 ± 0.67 in 1-hour images and less than 9.8 ± 0.45 in late images. After normalization to liver uptake of SUVmax values in tumor, kidneys, and shoulder, the highest SUVmax was observed in GBM (11 ± 2.5%), which was remarkably higher than the reference shoulder (3.4 ± 0.5%) with statistically significant difference (*p* < 0.001).

### 3.2. Therapy Studies

#### 3.2.1. Copper Inhibits Activity in Tumor Cell Population toward Neurosphere Formation

**Figure 2:**
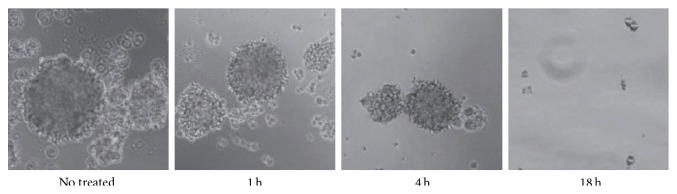
Cell neurospheres, photographed at 40 magnification, after 1, 4, and 18 h of exposure to ^64^Cu radiation. The neurosphere-forming ability of GBM cell lines was progressively abolished.

A typical behavior of U-87MG cell lines is their tendency to coalesce in CSC medium to form globular structures called neurospheres (Figure 2). This activity could be viewed as a prerequisite for cancer cells to grow a tumor mass. It was observed that, after 18 h exposure to [^64^Cu]CuCl_2_, the neurosphere-forming ability of GBM cell lines was completely suppressed.

#### 3.2.2. Treatment Response

**Figure 3:**
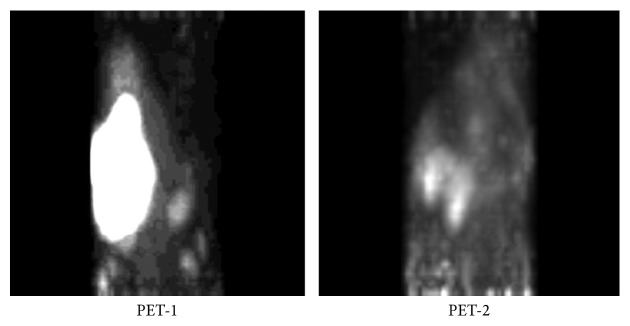
Representative PET image of MDG mouse number 6 showing 92% tumor volume reduction.

The tumor volume reduction from PET-1 to PET-2, expressed as percentage, for animals treated in SDG and MDG, is reported in Table 1. A good response to both single- and multiple-dose treatment was observed in almost all cases. In SDG, VOI reduction ranged from 68 to 94%, with complete tumor disappearance observed in two cases. In MDG, VOI reduction ranged from 64 to 92%, with complete tumor disappearance observed in four cases (Table 1, Figure 3).

Survival results among the three groups (Table 2) showed a significant increase in survival rates in SDG and MDG as compared to CG (*p* < 0.005). All the CG mice deceased within the 8th week, while in the MDG and SDG the first mice decease occurred starting from the 12th to 14th week, respectively. At the 20th week the survival rate in MDG and SDG was 70% and 73.3%, respectively. However no significant difference between the results of single- and multiple-dose administration was found.

#### 3.2.3. Radiotoxicity

SDG displayed a transient depression in white blood cell count (approximately, 40% average loss), but a normal value was recovered within 5 weeks. Liver and kidney functions remain unaltered while a loss of appetite was noted until 5-6 days after the injection with a moderate weight loss that recovered to normal levels after several days and retaining normal growth patterns.

While in MDG no symptoms of radiotoxicity were observed and the mean-weight increased being almost similar to the CG, the MDG animals maintained healthy physical appearance and the same blood test values as in CG over all the experimental period.

## 4. Discussion

Conventional design of targeted radiopharmaceuticals for molecular imaging and therapy commonly pursues the strategy of labeling a bioactive moiety, showing selective affinity for a specific biological substrate, with some suitable radionuclide.

Even ^64^Cu has been widely used to label peptides, proteins, antibodies, nanoparticles, and other biologically relevant small molecules through a variety of bifunctional chelators, such as DOTA [4]. Copper complexes have been successfully employed to develop a few target-specific radiopharmaceuticals although this approach suffers from many technical challenges connected with the requirements to achieve high specific activities, low stability in vivo, transchelate to serum proteins, or display poor acid stability [15].

Consequently, studies have investigated the potential role of the nonchelated ^64^Cu in its simplest chemical form as dichloride salt and its ability to selectively target cancerous cells for both imaging and therapy. Compared with ^64^Cu-labeled complex, [^64^Cu]CuCl_2_ has several advantages: easily available and clinically versatile, no complex radiolabeling process involved, high stability, and high bioactivity toward CTR1 [4]. Actually, an increasing number of recent publications have convincingly reported strong experimental evidence that ^64^Cu ions accumulate in various tumors including prostate, breast cancer, and melanoma [2–8]. It seems also well demonstrated that this ability of copper ions to enter cancer cells is not only receptor mediated, but also tightly connected with the metabolic role of this metal in cellular replication and in assisting tumor growth [14].

Following the same research approach, the present paper was aimed at extending the search of different tumor types that could potentially exhibit a similar enhanced affinity for [^64^Cu]Cu^2+^ ions. More precisely, our animal study investigated the biodistribution of [^64^Cu]CuCl_2_ in mice implanted with U-87MG glioblastoma-astrocytoma cell lines as a model of GBM, one of the most harmful malignancies of the central nervous system. It is worthy to note that to our knowledge this study is the first attempt to provide experimental evidence of the ^64^Cu uptake by GBM cells, for both diagnosis and treatment.

Taking advantage of the ^64^Cu positron emission and subsequent annihilation into two *γ* photons, we were able to collect images of its “in vivo” biodistribution by a small-animal PET scanner. Our preliminary results undoubtedly revealed that glioma cells, as found for other cancers, avidly incorporate ^64^Cu after its intravenous injection as dichloride salt.

Biodistribution revealed no brain uptake, with significant uptake in the liver and less in the kidneys 1 hour after injection, followed by a prevalent intestinal washout 20 hours after injection, suggesting that [^64^Cu]CuCl_2_ is mainly metabolized through hepatobiliary, gastrointestinal, and kidney systems. Evidently, this points out to the conclusion that, after injection in the blood pool, ^64^Cu ions are not freely diffusing but are likely linked to various proteins, which transport them to the target biological sites [13, 14] or can bind with superoxide dismutase, which is distributed widely in the cytosol of eukaryotic cells and abundant in the liver and kidney [4].

Moreover, our data demonstrated that the semiquantitative analysis, by using SUVmax, might support the visual interpretation of the PET images. SUVmax, after normalization to liver uptake, resulted remarkably higher in GBM than in the reference region with statistically significant difference.

On the other hand, we utilized the associated electron emission generated by the *β* ^−^ weak decay and electron capture of ^64^Cu to investigate the therapeutic potential of this new agent for the treatment of GBM. Interestingly, our preliminary results showed that a therapeutic dose of [^64^Cu]CuCl_2_ led to a significant progressive decrease and, in some cases, complete regression of tumor volume, thus suggesting an effective therapeutic role for [^64^Cu]CuCl_2_. In particular, we found a significant increase in the survival rate of the treated mice compared with those of the control group, even if any statistical significant differences were found between the two therapeutic protocols.

Our data about the radiotoxicity showed that there were no significant side effects on animals after ^64^Cu administration and, if present, they were completely reversible. It should be emphasized that chemical toxicity is usually not a concern with radiometals employed for nuclear imaging and radionuclide therapy since nanomolar amounts of the radioactive agent are normally injected, which fall well below the known toxicity limit for metallic species and, more specifically, for copper ions.

## 5. Conclusions

This study is the first report, to our knowledge, which demonstrates that [^64^Cu]CuCl_2_ exhibits enhanced affinity for GBM cells with statistically significant increase of tracer uptake as compared to normal cells, thus supporting its potential role as a novel promising diagnostic PET probe for cerebral tumor imaging. Furthermore, our results on therapy utilization suggest that this new radiopharmaceutical has a great potential as therapeutic agent for GBM as clearly showed by survival results in experiments carried out with single- and multiple-dose treatments. These promising results deserve further investigation in preclinic and clinic studies.

## Figures and Tables

**Table 1:** Tumor volume reduction from PET-1 to PET-2 (expressed as percentage) in single-dose group (SDG) and multiple-dose group (MDG), respectively.

Mouse	SDG^*∗*^ (%)	MDG^*∗*^ (%)
1	93	78
2	O	X
3	91	71
4	86	64
5	X	69
6	68	92
7	O	X
8	80	O
9	73	73
10	70	X
11	X	X
12	84	X
13	91	80
14	X	O
15	68	88
16	73	75
17	79	84
18	84	X
19	72	69
20	X	X
21	88	92
22	90	X
23	72	O
24	X	88
25	94	X
26	X	O
27	X	76
28	77	75
29	75	82
30	X	90

^*∗*^X = mouse deceased; O = tumor disappearance.

**Table 2:** Survival results for the three animal groups.

Time (weeks)	CG^*∗*^	SDG^*∗*^	MDG^*∗*^
1°	2		
2°	3		
3°	3		
4°	3		
5°	5		
6°	4		
7°	5		
8°	5		
9°			
10°			
11°			
12°			2
13°			
14°		2	2
15°		2	
16°		2	1
17°			1
18°			3
19°			
20°		2	
**Total**	**30/30**	**8/30**	**9/30**

^*∗*^Number of mice deceased.
